# Hypothalamic-Pituitary-Adrenal Axis Programming after Recurrent Hypoglycemia during Development

**DOI:** 10.3390/jcm4091729

**Published:** 2015-08-28

**Authors:** Raghavendra Rao

**Affiliations:** 1 Department of Pediatrics, Division of Neonatology, University of Minnesota, Minneapolis, MN 55455, USA; E-Mail: raghurao@umn.edu; Tel.: +1-612-625-3260; Fax: +1-612-624-8176; 2 Center for Neurobehavioral Development, University of Minnesota, Minneapolis, MN 55455, USA

**Keywords:** brain injury, cortisol, corticosterone, development, hypoglycemia, hypoglycemia-associated autonomic failure, hypothalamus-pituitary-adrenal axis, programming, recurrent hypoglycemia

## Abstract

Permanent brain injury is a complication of recurrent hypoglycemia during development. Recurrent hypoglycemia also has adverse consequences on the neuroendocrine system. Hypoglycemia-associated autonomic failure, characterized by ineffective glucose counterregulation during hypoglycemia, is well described in children and adults on insulin therapy for diabetes mellitus. Whether recurrent hypoglycemia also has a programming effect on the hypothalamus-pituitary-adrenal cortex (HPA) axis has not been well studied. Hypoglycemia is a potent stress that leads to increased glucocorticoid secretion in all age groups, including the perinatal period. Other conditions associated with exposure to excess glucocorticoid in the perinatal period have a programming effect on the HPA axis activity. Limited animal data suggest the possibility of similar programming effect after recurrent hypoglycemia in the postnatal period. The age at exposure to hypoglycemia likely determines the HPA axis response in adulthood. Recurrent hypoglycemia in the early postnatal period likely leads to a hyperresponsive HPA axis, whereas recurrent hypoglycemia in the late postnatal period lead to a hyporesponsive HPA axis in adulthood. The age-specific programming effects may determine the neuroendocrine response during hypoglycemia and other stressful events in individuals with history of recurrent hypoglycemia during development.

## 1. Introduction

Hypoglycemia is the most common metabolic problem in infants and children. The common causes of recurrent hypoglycemia during this period are congenital hyperinsulinism, inborn errors of carbohydrate, amino acid and lipid metabolism, and insulin therapy for type 1 and type 2 diabetes. Severe and recurrent hypoglycemia leads to permanent brain injury. Therefore, most studies of hypoglycemia in infants and children have focused on brain structural changes and functional outcomes (reviewed [1]). However, hypoglycemia has detrimental effects beyond brain injury. Hypoglycemia-associated autonomic failure (HAAF), which is characterized by defective glucose counterregulation during hypoglycemia, is a well-known neuroendocrine complication in children and adults on insulin therapy for type 1 and type 2 diabetes [2,3]. The pathogenesis of HAAF is not fully understood; however, an attenuated adrenal medullary response to hypoglycemia is a major component [2,4]. A detailed discussion of HAAF is beyond the scope of this review. Excellent recent reviews on pathogenesis and treatment of HAAF are available elsewhere [2,3,4]. Instead, this review will focus on the programming effects of recurrent hypoglycemia on the hypothalamic-pituitary-adrenocortical (HPA) axis. The HPA axis plays a critical role in development and physiology in all mammals, including humans. A normally functioning HPA axis is important for optimal health. Early life stress programs the HPA system and determines how an individual responds to stress later [5,6,7]. The long-term effects of recurrent hypoglycemia on the developing HPA axis are not well known, despite hypoglycemia being one the most potent stressors. In the following sections, hypoglycemia-induced brain injury during development, age-related variations in the vulnerability to this injury, the normal HPA axis response during hypoglycemia, and the long-term effects of recurrent hypoglycemia on the HPA axis are reviewed. Most of the data are from animal studies using rat models. Rats are commonly used for determining the effects of hypoglycemia on the developing brain [8,9,10], as well as to study HPA axis programming in perinatal stress [7,11]. 

## 2. Hypoglycemia-Induced Brain Injury during Development

Glucose is the primary energy substrate to the brain at all ages. The brain has minimal carbohydrate stores in the form of brain glycogen. Thus, a constant supply of glucose from plasma to the brain is necessary for maintaining cerebral energy production. Neuroglycopenia during hypoglycemia leads to cellular energy failure and initiates neuronal injury through a multi-step process [1,12,13]. Extensive neuronal death and astrocytosis in all brain regions, except the cerebellum, is a hallmark of severe hypoglycemia that is associated with coma or seizures (typical plasma glucose concentration <1.1 mmol/L (<20 mg/dL)) [14]. However, such severe hypoglycemia is rare in clinical practice [15]. Most hypoglycemia episodes during development are of mild to moderate severity (typical plasma glucose concentration 1.1–2.5 mmol/L (20–45 mg/dL)) and are not associated with coma or seizures. Studies in developing rats demonstrate that moderate hypoglycemia leads to neuronal injury confined primarily to the orbital and cingulate regions of the frontal cortex and layers 2 and 3 of the parietal and temporal cortices [8,9,10,13,16]. Other brain regions, including the hypothalamus, are spared. Altered hippocampal synaptogenesis (unpublished observations) and electrophysiological dysfunction without detectable hippocampal neuronal injury [9] have also been reported following recurrent hypoglycemia in three-week-old rats (neurodevelopmentally equivalent to a young child). A similar regional distribution of injury is seen after acute and recurrent moderate hypoglycemia in adult rats [8,13,17,18]. The animal studies further demonstrate that the developing brain is more resistant to injury during an acute episode of moderate hypoglycemia, relative to the mature brain [8,13]. This could be due to lower cerebral energy requirement, higher antioxidant concentrations in the brain regions and a greater capacity to use alternative energy substrates, such as ketone bodies, lactate and brain glutamate and glutamine during development [19,20,21,22]. However, an opposite effect is likely when hypoglycemia is recurrent. The same adaptive responses that protect the developing brain during hypoglycemia may lead to altered neurodevelopment, given the necessity of glutamatergic stimulation during synaptogenesis. Recurrent moderate hypoglycemia in developing rats is associated with immediate and long-term functional deficits [9,10], whereas neither immediate nor long-term effects are the sequelae of recurrent hypoglycemia in adult rats [23,24]. Similarly, studies in children and adults with type 1 diabetes demonstrate that recurrent hypoglycemia before five years of age negatively affects cognitive function [25], whereas no such effects are seen with recurrent hypoglycemia in adolescents and adults [26].

## 3. Counterregulatory Hormonal Response during Hypoglycemia

The mechanisms that prevent and/or rapidly correct hypoglycemia are (1) decreased pancreatic β cell insulin secretion, (2) increased pancreatic α cell glucagon secretion and (3) increased adrenal medullary catecholamine secretion. The cumulative effect of these adaptive hormonal responses is suppression of tissue glucose utilization and improved glucose availability through hepatic glycogenolysis and gluconeogenesis. Additionally, there is increased secretion of cortisol from the adrenal cortex and growth hormone from the anterior pituitary. The latter hormonal responses are relatively delayed and do not correct hypoglycemia *per se*, but rather contribute to glucose counterregulation by shifting non-central nervous system (CNS) tissue metabolism away from glucose utilization. The peripheral and central neural pathways that control glucose counterregulation is beyond the scope of this review and excellent reviews available elsewhere [27,28,29]. In addition to the hormonal response, hypoglycemia leads to catecholamine-mediated autonomic symptoms (palpitations, tremor and anxiety), whose function is to increase arousal and elicit feeding and thereby hasten correction of hypoglycemia. Children have a more robust catecholamine response and become symptomatic at a higher glycemia threshold than the adults (plasma glucose concentration, 3.6–4.2 mmol/L (65–75 mg/dL) *vs.* 3.0 mmol/L (54 mg/dL) [30]. Conversely, newborn infants are able to tolerate lower plasma glucose levels (<2.7 mmol/L (<45 mg/dL)) without exhibiting counterregulatory hormonal response or symptoms [31]. A greater ability to use alternative substrates, such as ketone bodies and lactate, for energy production may be responsible for this. An inappropriately low plasma cortisol and adrenocorticotropic hormone (ACTH) concentrations due to lack of hypothalamic-pituitary axis drive is often present during hyperinsulinemic hypoglycemia in the newborn period [32,33,34]. 

## 4. Hypoglycemia and the HPA Axis

### 4.1. HPA Axis-Components and Normal Response

Stressors such as hypoglycemia induce the release of corticotropin-releasing hormone (CRH) from the hypothalamic paraventricular nucleus (PVN). CRH acts on the anterior pituitary to stimulate ACTH synthesis from proopiomelanocortin (POMC). ACTH, in turn, stimulates cortisol (corticosterone in rodents) secretion from the adrenal cortex. HPA activity is inhibited by negative feedback of glucocorticoids at the mineralocorticoid receptors (MR) in the hippocampus, and the glucocorticoid receptors (GR) in the hippocampus, hypothalamus and pituitary [35]. The hippocampal MR and GR are most sensitive to circulating corticosterone between postnatal day (P) 18 and P28 in rats [36].

### 4.2. Age-Related Variations in HPA Axis Response to Hypoglycemia

In humans, ACTH bioactivity in the pituitary appears at approximately 25% of the total gestation (at eight weeks). In rats, it appears only at around 75% of the gestation (on gestational day 16, normal gestation = 21–22 days) [37,38]. Thus, the HPA axis is more mature at birth in humans, relative to rats. As mentioned, plasma cortisol concentration during hyperinsulinemic-hypoglycemia is inappropriately low in human newborn infants [32,33,34]. A poor hypothalamic-pituitary drive, rather than immaturity of the adrenal cortex, is responsible for this, since the cortisol response to ACTH administration is intact [32,33,34]. A period of relative HPA axis insensitivity (“stress hyporesponsive period”) during the first two postnatal weeks is a well-established fact in rats and mice. The hyporesponsiveness is context and stimulus specific. Basal CRH secretion from the hypothalamus in rats younger than 35 days is only 16%–42% of that in adult rats [39]. No additional increase in CRH secretion is seen during hypoglycemia. Ten-day-old rats (neurodevelopmentally equivalent to a human term newborn infant) do not mount a robust corticosterone response to insulin-induced hypoglycemia, despite increased ACTH secretion, suggesting that the defect is at the level of adrenal cortex [39]. However, an inability of the hypothalamus to secrete CRH may be also responsible [39,40]. Any ACTH and corticosterone response to hypoglycemia in the neonatal period appears to be mediated by arginine vasopressin (AVP) and not CRH [41]. A robust HPA axis response to hypoglycemia is seen only after 19 days of age (*i.e.*, near weaning time) in rats [39]. A stress hyporesponsive period also likely exists in human infants and begins at 12 months of age [42]. The duration of this hyporesponsive period has yet to be determined. However, both preterm and full-term infants are capable of mounting robust cortisol response in the newborn period, suggesting a mature HPA axis at birth.

### 4.3. Recurrent Hypoglycemia and the HPA Axis

Even a single episode of antecedent hypoglycemia alters the neuroendocrine response during subsequent hypoglycemia [18,43]. This condition known as HAAF or hypoglycemia unawareness (because of the absence of catecholamine-mediated physical symptoms) is a known complication of insulin therapy for type 1 and 2 diabetes. Although the mechanism of HAAF is not fully understood, improved brain glucose transport is a major factor [44]. Brain glucose concentration is higher in adult humans with type 1 diabetes and HAAF, relative to the controls [45]. Chronic and recurrent hypoglycemia enhances blood brain glucose transport capacity in adult rats [46,47]. Increased expression of glucose transporters at the blood-brain barrier also has been demonstrated in developing rats after recurrent hypoglycemia [48]. HAAF is characterized by lack of suppression of endogenous insulin secretion and failure of glucagon and catecholamine secretion during hypoglycemia. Decreased cortisol secretion is commonly present; however, adrenal medullary effects predominate. Increased CRH secretion, acting via CRH receptor 1, may be involved in the sympathoadrenal downregulation [49]. Whether HAAF-equivalent neuroendocrine effects, especially adrenocortical effects, also occur during a non-hypoglycemic stress has yet to be determined.

### 4.4. Recurrent Hypoglycemia and HPA Axis Programming

The HPA axis is susceptible to programming in the perinatal period. A variety of prenatal and postnatal stressors are known to program the HPA axis to either hyper- or hypo-respond to stimuli in later life [7,11]. Maternal malnutrition, maternal stress and exposure to hypoxia or synthetic glucocorticoids in the prenatal period, maternal separation, inadequate stimulation, malnutrition and recurrent hypoxia in the postnatal period are the common perinatal conditions known to program the HPA axis [7,11,50]. Exposure to excess glucocorticoids, either stress-induced or exogenously administered, in the perinatal period is likely responsible for the HPA axis programming. Oxidative stress and genetic and epigenetic alterations in glucocorticoid receptors are potentially involved in the programming [7,51].

Hypoglycemia is a potent stressor that causes increased cortisol secretion. Even 8-day-old neonatal rats mount ACTH and corticosterone response during insulin-induced hypoglycemia, although the response is not as robust as in older rats (P19 and beyond) [39,41,52]. Further, the response in the neonatal period likely involves AVP, instead of CRH [40]. Maternal separation augments hypoglycemia-induced corticosterone response in the early postnatal period [52]. Hypoglycemia is also associated with oxidative stress, especially in the post-hypoglycemia period [13,19,21,53,54]. Thus, recurrent hypoglycemia is associated with at least two factors (excess glucocorticoids and oxidative stress) involved in the pathogenesis of HPA axis programming [7,51].

Two studies in developing rats have determined the long-term effects of recurrent hypoglycemia on the HPA axis and stress reactivity. In the first study, Moore and colleagues [10] induced insulin-induced moderate hypoglycemia (blood glucose concentration, 1.6–2.2 mmol/L (30–40 mg/dL) for approximately 3 h) twice daily from P10 to P19 (20 episodes) [39]. The control group received normal saline. Neuronal injury was assessed at various time points between P23 (subacute period) and P51 (adulthood). A variety of behaviors (maternal separation-induced ultrasonic vocalization, social play behavior, habituation of the acoustic startle response, prepulse inhibition of startle, fear-potentiated startle) were tested during the period of recurrent hypoglycemia (P9–P14), in the subacute period (P22–P34) and at adulthood (P55–P60). The behavioral and hormonal response to a 30 min restraint stress was also assessed in adulthood [10]. 

Recurrent hypoglycemia led to mild neuronal injury in the parasagittal and temporal cortices, similar to a previous acute hypoglycemia study at the corresponding age [8]. Animals subjected to recurrent hypoglycemia demonstrated increased maternal separation-induced ultrasonic vocalization and decreased habituation of the acoustic startle response in the subacute period. Social play behavior during adolescence was decreased. Exaggerated acoustic response and fear-potentiated startle persisted in adulthood. Stress-induced facial tremors were increased during restraint stress in adulthood and there was a trend towards increased stress-induced plasma corticosterone response in males, but not in females [10]. Collectively, these effects suggest increased anxiety and a hyperresponsive HPA axis, similar to the response after other stressors in the early postnatal period [10]. Unfortunately, the corticosterone response during hypoglycemia and the baseline corticosterone concentration in adulthood were not assessed in the study [10].

A second study from our lab assessed the long-term effects of recurrent hypoglycemia in the late postnatal period on the HPA axis activity at adulthood [55]. Three-week-old rats were subjected to five episodes of insulin-induced moderate hypoglycemia (blood glucose concentration, 1.7 ± 0.7 mmol/L (31 ± 15 mg/dL)), once daily from P24 and P28. This hypoglycemia model results in neuronal injury in the prefrontal, parietal and temporal regions of the cerebral cortex and decreased dendritic arborization without evident neuronal injury in the hippocampus (unpublished data). Brain and adrenal glands were harvested on P90 (adulthood). The GR and MR transcript expression in the hippocampus, hypothalamus, pituitary and adrenal cortex, and the CRH and AVP transcript expression in the hypothalamus were determined. The harvested adrenal cortical cells were stimulated with ACTH to determine the corticosterone response. Some rats were subjected to 30 min of restraint stress on P90 and the serum corticosterone concentration before and after the stress was determined [55]. 

Compared with the control group, CRH transcript expression in the hypothalamus was decreased at adulthood in rats subjected to recurrent hypoglycemia. AVP transcript expression was not altered. In the adrenal gland, the transcript expression of ACTH receptor and low-density lipoprotein receptor (LDLR) necessary for cholesterol uptake from the plasma was lower. The expression of GR and steroidogenic acute regulatory protein (StAR) necessary for mitochondrial cholesterol uptake for steroidogenesis was higher. The expression of other steroidogenic enzymes was not altered. StAR and corticosterone response to ACTH stimulation was blunted in the adrenal cortical cells. Similarly, the post-restraint corticosterone response was blunted, despite comparable baseline corticosterone concentration as the control group. The expression of GR and MR transcripts in the hippocampus was increased, likely in response to the decreased corticosterone. GR, MR and POMC transcript expression in the pituitary was not altered [55]. Collectively, these data suggest that recurrent hypoglycemia in the late postnatal period leads to a hyporesponsive HPA axis in adulthood. The lower CRH expression suggests that the effects are likely mediated at the level of the hypothalamus. The hypothalamic PVN activity is regulated by many G-protein-coupled receptors [56]. Downregulation of any of these receptors may have led to dampened CRH expression and hyporesponsive HPA axis [57]. However, the lower LDLR transcript expression and the blunted StAR response to ACTH stimulation in the adrenal cortical cells suggest the possibility of a programming effect at the level of the adrenal cortex also. Whether these effects were sex-specific and were mediated by increased plasma corticosterone during antecedent hypoglycemia was not assessed in the study. Similarly, whether the neuroendocrine effects were limited to the adrenal cortex or also involved the adrenal medulla (lack of sympathoadrenal response, similar to HAAF) was not determined [55].

Although experimental variations preclude direct comparison between the two studies, some general conclusions can be drawn. The programming effect of recurrent hypoglycemia on the HPA axis appears to be influenced by its timing in relation to the stage of development. Recurrent hypoglycemia in the early postnatal period (*i.e.*, during the stress hyporesponsive period) potentially leads to a hyperresponsive HPA axis in adulthood, whereas recurrent hypoglycemia in the late postnatal period (*i.e.*, beyond the stress hyporesponsive period) likely leads to a hyporesponsive HPA axis, at least in male rats. These effects parallel the effects of other stressors at corresponding ages. For example, exposure to lipopolysaccharide (LPS), a potent HPA axis stimulant, in the neonatal period leads to increased HPA axis activity and anxiety-like behaviors in adulthood [58], whereas recurrent LPS administration in adulthood leads to a hyporesponsive HPA axis [59]. A similar time-dependent effect of stress on HPA axis sensitization has also been demonstrated in a large prospective trial of human adolescents [60]. Determination of salivary cortisol after a social stress test in 16-year-old adolescents demonstrated that exposure to stress in childhood (<11 years) is associated with cortisol hypersecretion, whereas exposure to stress in early and middle adolescence (>11 year) is associated with cortisol hyposecretion [60]. 

## 5. Clinical Implications 

Inter-species differences in substrate utilization and HPA axis maturation between developing rats and human infants preclude direct extrapolation of the animal data to human infants and children without definitive experiments. Preweaning rats are naturally ketogenic and capable of utilizing ketone bodies for energy production [61]. Thus, they may be able to withstand low plasma glucose (<3.0 mmol/L (<54 mg/dL)) without mounting counterregulatory catecholamine response [62]. Conversely, healthy human children exhibit neurological dysfunction and counterregulatory hormonal response at higher blood glucose concentrations (3.6–4.2 mmol/L (65–75 mg/dL)) [30]. Relative to rats, the HPA axis is more mature at birth in human infants [37,38]. Despite these caveats, the results of the animal studies may have clinical implications. A hyperresponsive HPA axis as a consequence of recurrent hypoglycemia in the early postnatal period may improve the ability to withstand hypoglycemia in later life. Male rats subjected to recurrent hypoglycemia from P9 to P20, a period that compares with the period of hypoglycemia in the Moore *et al.* study [10]), were able to tolerate hypoglycemia better in adulthood than those that were not exposed to hypoglycemia during development [63]. A similar effect was not present in the female rats, suggesting a sex-specific programming effect. However, the putative advantage of being able to withstand hypoglycemia (and presumably other stressful conditions) is offset by the increased risk of developing a wide range of cardiovascular, psychiatric and metabolic disorders that have been described in the context of both, hyper- and hypo-responsiveness to stress [64,65]. Moreover, a hyporesponsive HPA axis as a sequela of recurrent hypoglycemia in the late postnatal period may preclude appropriate response during a stressful situation (e.g., social stress) [60] or hypoglycemia later in life. The latter is a major concern for those on intensive insulin therapy for type 1 and type 2 diabetes, since they are at risk for hypoglycemia throughout life.

## 6. Conclusions

This review provides evidence that recurrent hypoglycemia during development has long-term adverse effects on the HPA axis activity. The timing of exposure to hypoglycemia in relation to the developmental stage likely determines whether the HPA axis hyperresponds or hyporesponds during subsequent hypoglycemic and non-hypoglycemic stresses. Future research should explore the mechanism of age- and sex-specific HPA axis programming in recurrent hypoglycemia, its potential reversibility and its effects on the brain regions and other organs.
